# A Validated Normative Model for Human Uterine Volume from Birth to Age 40 Years

**DOI:** 10.1371/journal.pone.0157375

**Published:** 2016-06-13

**Authors:** Thomas W. Kelsey, Eleanor Ginbey, Moti M. Chowdhury, Louise E. Bath, Richard A. Anderson, W. Hamish B. Wallace

**Affiliations:** 1 School of Computer Science, University of St Andrews, St Andrews KY16 9SX, United Kingdom; 2 School of Medicine, University of Edinburgh, Edinburgh EH16 4TJ, United Kingdom; 3 Department of Paediatric Radiology, Royal Hospital for Sick Children, Edinburgh EH9 1LF, United Kingdom; 4 Department of Endocrinology and Diabetes, Royal Hospital for Sick Children, Edinburgh EH9 1LF, United Kingdom; 5 MRC Centre for Reproductive Health, University of Edinburgh, Edinburgh EH16 4TJ, United Kingdom; 6 Department of Haematology/Oncology, Royal Hospital for Sick Children, Edinburgh EH9 1LF, United Kingdom; VU University Medical Center, NETHERLANDS

## Abstract

Transabdominal pelvic ultrasound and/or pelvic Magnetic Resonance Imaging are safe, accurate and non-invasive means of determining the size and configuration of the internal female genitalia. The assessment of uterine size and volume is helpful in the assessment of many conditions including disorders of sex development, precocious or delayed puberty, infertility and menstrual disorders. Using our own data from the assessment of MRI scans in healthy young females and data extracted from four studies that assessed uterine volume using transabdominal ultrasound in healthy females we have derived and validated a normative model of uterine volume from birth to age 40 years. This shows that uterine volume increases across childhood, with a faster increase in adolescence reflecting the influence of puberty, followed by a slow but progressive rise during adult life. The model suggests that around 84% of the variation in uterine volumes in the healthy population up to age 40 is due to age alone. The derivation of a validated normative model for uterine volume from birth to age 40 years has important clinical applications by providing age-related reference values for uterine volume.

## Introduction

In the adult human female the uterus is approximately the shape and size of a pear and sits in an inverted position within the pelvic cavity. The uterus is sited along the body’s midline posterior to the urinary bladder and anterior to the rectum and consists of a body and a cervix that protrudes into the vagina. The primary function of the uterus is to nourish and protect the developing fetus during pregnancy until birth.

In addition to normal somatic growth, the main change in the size of the uterus with age is at puberty, when it grows in response to endocrine stimulation and changes from tubular to ‘pear’ shaped [1]. The ratio of the corpus to cervix also changes during puberty from being roughly 1:1 before puberty, to between 2:1 and 3:1 after puberty [2]. The size of the uterus increases with parity [3], and can also be enlarged in various common pathological conditions such as the presence of leiomyomata (fibroids).

Transabdominal pelvic ultrasound and/or pelvic Magnetic Resonance Imaging (MRI) are safe, accurate and non-invasive techniques for determining the size and configuration of the internal genitalia in pre-pubertal, pubertal and adult females [4, 5]. Ultrasound provides a quicker examination (useful for younger/ non-compliant and claustrophobic patients) that is more readily available and accessible, whilst MRI visualisation of the adnexae is less susceptible to physiological conditions of incomplete bladder filling, bowel gas obscuration and is useful in patients with high body mass index where adequate sonographic visualisation is precluded, whilst providing for measurements that are less operator-dependent.

The assessment of uterine size and volume is helpful in the assessment of conditions including disorders of sex development (DSD), precocious puberty, absent menstruation with or without pubertal delay [1, 6], infertility, menstrual disorders, pelvic masses, ambiguous genitalia and in the survivor of childhood or adult cancer who has been exposed to radiotherapy to a field that includes the pelvis [7–9]. Previous studies of changes in uterine volume with age have been restricted to changes in uterine length, or uterine volume across small age ranges. In this study we have developed a validated normative model for healthy uterine volume from birth to age 40 years.

## Methods

The data for this study are from two sources: our own measurements of uterine volume from unpublished paediatric MRI scans, and data extracted from the published peer reviewed literature. The combined dataset (n = 1,418) forms a representative sample of uterine volumes for the healthy population from birth to age 40 years.

**Fig 1:**
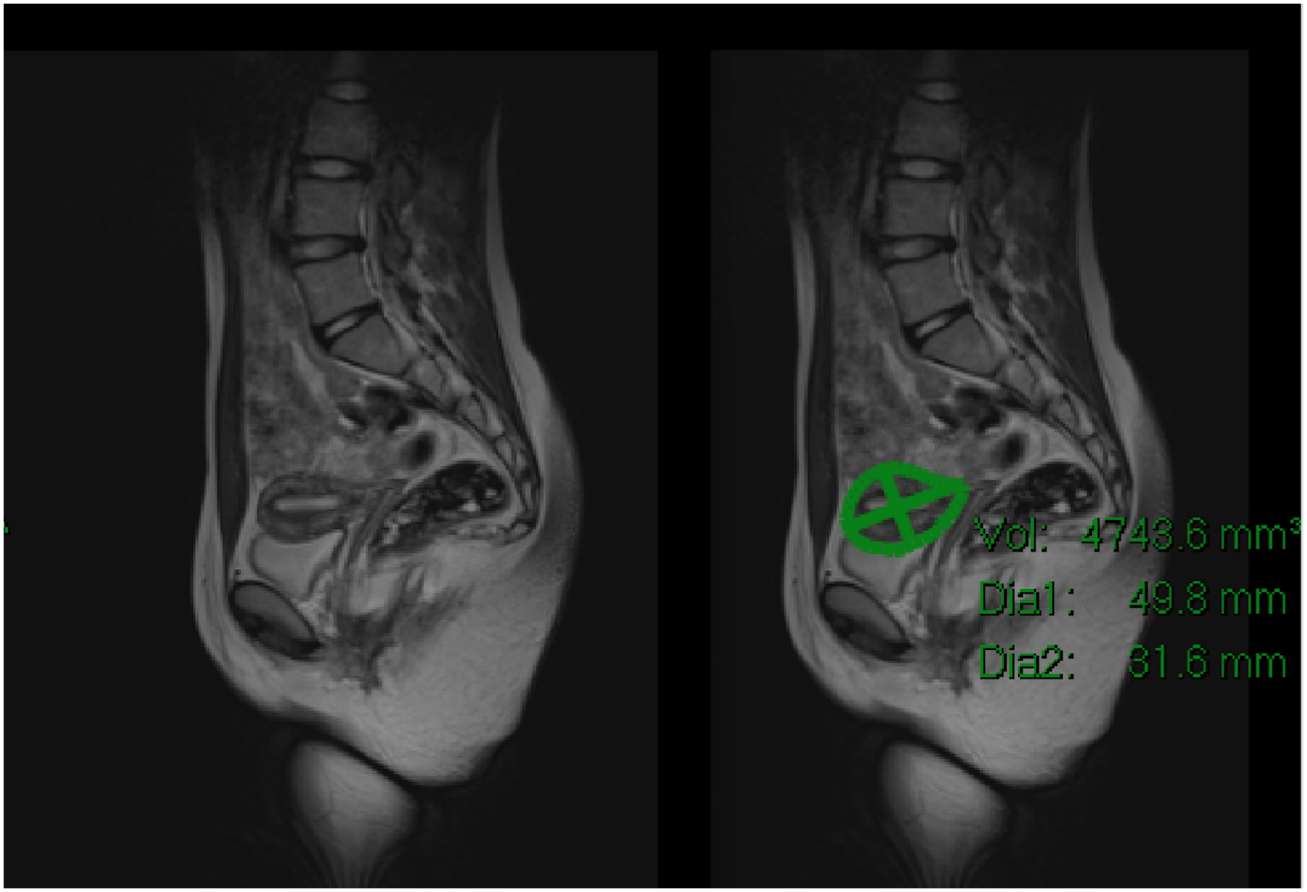
MRI pelvis with Sagittal T2 spin echo sequence demonstrating normal uterine morphology (left) with volume measurement (right) using a summation of area within the region of interest (ROI) from each contiguous image slice through the visualised uterus.

Uterine volumes were measured from paediatric patients who had MRI scans that included imaging of their pelvis, the same scans from which ovarian volume had been previously determined [10] (Fig 1). MRI scans were assessed from 120 children ages 0–16 (median 12.0; SD 4.8) years without known endocrine, congenital or oncological problems that included the pelvis. The scans were taken to elucidate possible hip abnormalities. From these 120 MRI scans, 20 were excluded due to the uterus being difficult to visualise (often in scans not taken with the sole purpose of imaging the pelvis), 8 were excluded due clarity compromised by motion artefact, and 5 were excluded due to the presence of suspected reproductive pathologies. Hence 87 uterine volumes were obtained, with only one scan per patient used and a scan excluded if the uterus could not be clearly visualised. The majority of scans were measured on T2 weighted spin-echo (SE) sequences, which provides optimal contrast resolution of uterine signal as seen in the left panel of Fig 1. For each plane the images are acquired contiguously, with each image being a pre-defined thickness ‘slab’. To measure the uterus, lesion segmentation tool on PACS (Picture Archiving Communication System) workstation was used (Fig 1, right panel). Internal PACS software calculates the area within the region of interest (ROI) that the radiologist circumscribes around the uterus and then multiplies this with the depth of the slab, to create a volume from the ROI drawn, for that segment of the uterus. By repeating this on each contiguous slab within which the uterus is identified, the software aggregates the volume of each slab to provide a final uterine volume. In patients where the uterus was reliably measurable on more than one plane (sagittal/ coronal/ axial), a mean uterine volume was obtained following measurements within each plane. Whilst the sagittal plane most naturally outlines the uterus morphology and mirrors the equivalent view seen on ultrasound assessments, body MRI predominantly utilises axial and coronal plane imaging for conventional anatomical assessment. Given this, in the majority of cases where sagittal plane imaging was not available, most measurements were obtained from coronal plane imaging, which usually provided superior visualisation of the uterus compared to axial imaging. Intra-observer error was assessed by calculating the variability between the volumes obtained by two observers, each blinded to the other’s measurements. If there was above 10% variability per scan, measurements were repeated to ensure no values were over or underestimated.

The second data set was extracted from published literature using an established methodology [10–14]. Pubmed, Medline and Embase were searched using the terms ‘normative’ ‘uterus’ and ‘ultrasound’. References of the identified studies were then retrieved and any other relevant research papers were extracted. Papers were included if they contained data from healthy, normal girls with no pelvic endocrine, congenital or reproductive problems to ensure that the data are representative of the healthy population. Abstracts of 15 papers were identified this way. Any subjects who were not healthy or were listed by Tanner stages of puberty instead of age were excluded. Papers reporting uterine length as opposed to uterine volume were also excluded. Of these 15 papers, there were 4 extractable sets of data for uterine volume obtained by transabdominal ultrasound [4, 5, 15, 16] (Table 1), 10 cases of data being reported as descriptive statistics and hence not suitable for digital extraction [17–26], and 1 case of data consisting of uterine length only [27], (Table 2). In all the studies used for data extraction the uterine volume was calculated using the modified formula for the prolate ellipsoid (0.5 X Length X Height X Width). These data were extracted using plot digitiser software [28] to convert data points from the scatter graphs into numerical data. Table 1 summarises the quantity, age range and source for each component of the combined dataset. Data were independently extracted by two observers to guard against miscalibration, with data used from the extracted data that had the closest match to the descriptive statistics reported in the supporting publication. In particular, the data in [15] were plotted with a high degree of overlap. Our extracted data from this source has correlation coefficient 0.50 and linear regression equation coefficients 0.14 and 12.9; the descriptive statistics are correlation coefficient 0.52 and linear regression equation coefficients 1.84 (clearly a typographic error) and 13.05.

**Table 1:** Uterine volume data summary. Pubmed ID, first author and year of publication are given for sources of extracted data, together with number of measurements and age ranges.

PMID	First author	Year	N	Median age (range)
20572067	Razzaghy-Azar	2010	240	9.6 (6.2–13.6)
14962602	Gadhela de Costa	2004	828	18.2 (9.6–49.3)
18951545	Badouraki	2008	99	7.0 (1.0–11.0)
8521066	Holm	1995	164	14.2 (6.4–25.4)
	Own data		87	11.9 (0.3–16.7)
	**Total data**		**1418**	**14.8 (0.3–49.3)**

**Table 2:** Excluded uterine volume data summary. Pubmed ID, first author and year of publication are given for sources of non-extracted data, together with number of measurements. Each publication met the inclusion and exclusion criteria for consideration as a data source, but either contained descriptive statistics of uterine volume rather than extractable data in the form of scatter plots, or data on uterine length (rather than volume).

PMID	First Author	Year	N	Why excluded
10746894	Cagnacci	2000	48	Descriptive stats
12020983	Christodoulacos	2002	23	Descriptive stats
8008485	Haber	1994	188	Descriptive stats
10206214	Haber	1999	190	Descriptive stats
12418765	Herter	2002	90	Uterine length only
3883910	Salardi	1985	114	Descriptive stats
8282889	Rigsby	1994	125	Descriptive stats
16703306	Hauth	2007	100	Descriptive stats
16728550	de Vries	2006	103	Descriptive stats
7758513	Haber	1995	55	Descriptive stats
3286855	Salardi	1998	133	Descriptive stats

Given that the volume of the uterus is always zero at time of conception, we predicted uterine volume from this time point by adding zero volumes at conception to the combined dataset. Box-Cox analysis indicated that the data should be log transformed. We then fitted 475 mathematical models to the data using TableCurve-2D (Systat Software Inc., San Jose, California, USA), and ranked the results by coefficient of determination, r^2^. Each model defines a generic type of curve and has parameters which, when instantiated gives a specific curve of that type. For each model we calculated values for the parameters that maximise the r^2^ coefficient. The Levenberg-Marquardt non-linear curve-fitting algorithm was used throughout, with convergence to 9 significant figures after a maximum of 4,000 iterations, for models having up to 21 parameters. For each candidate model, the mean square error and r^2^ were calculated after removing the artificial zero values at conception. In addition LOESS regression was used to investigate the possibility that the best predictive model may be an ensemble of locally linear or quadratic models, rather than a single model covering all age ranges.

**Fig 2:**
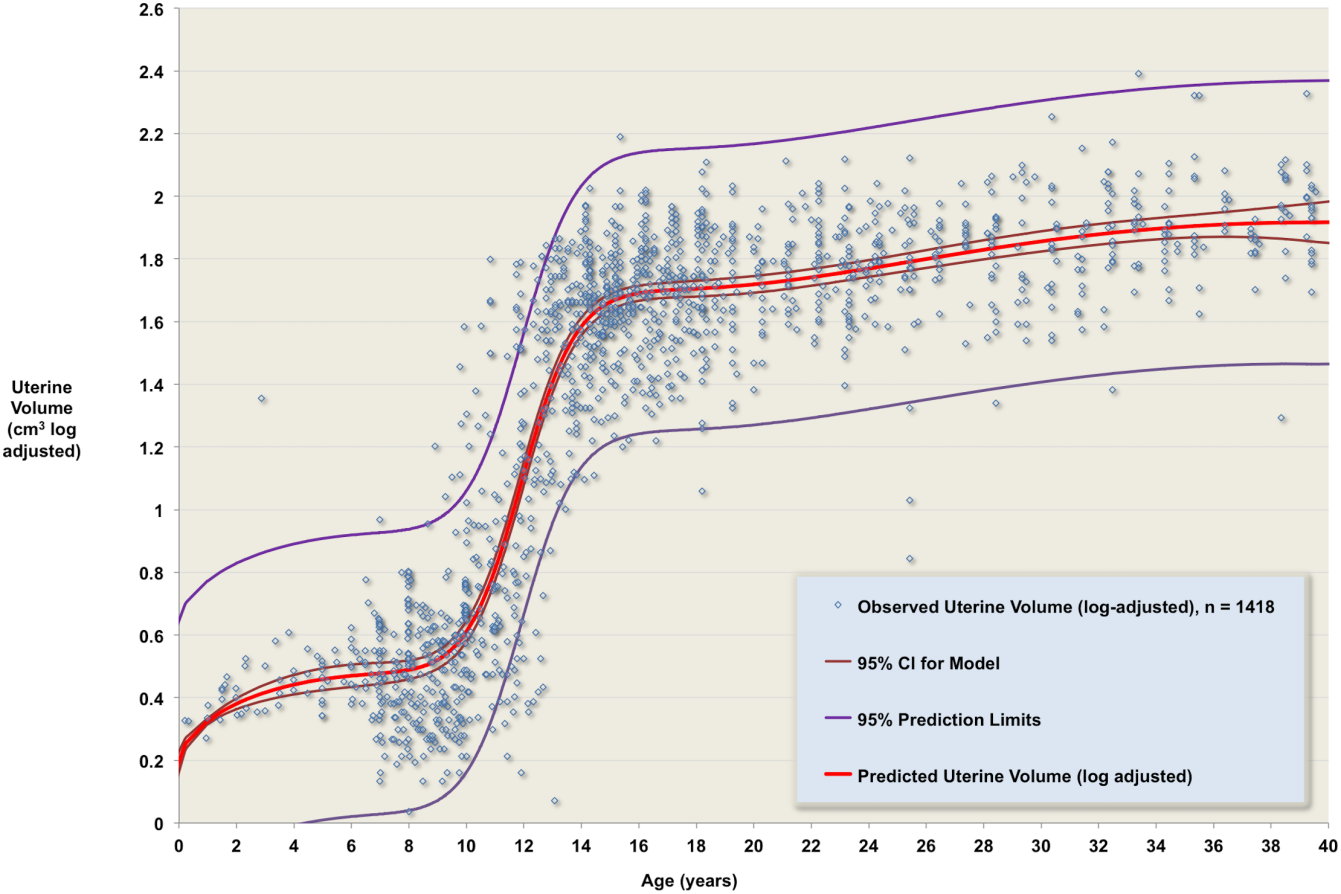
Log-adjusted data and normative model. The observed volumes (blue points) are shown with the predicted volume (red line), the 95% confidence interval for the predicted volume (brown lines), and the 95% prediction limits for the model (purple lines).

The best performing model was a ten-parameter rational polynomial (Fig 2, Table 3). 4-fold cross validation was performed to guard against the possibility that the optimal model was obtained by a serendipitous combination of initial data. The data were randomly split into 4 equally sized subsets, S1 –S4, each having approximately equal descriptive statistics. At the i-th stage, Si is removed from the dataset, the candidate model is fitted to the remaining three subsets (giving ten new parameters in each case), and the mean square error is calculated for the Si data (which wasn’t used to derive the new parameters). These four mean square errors were then compared to the mean square error for the whole dataset (Table 4). A model was considered validated if

**Fig 3:**
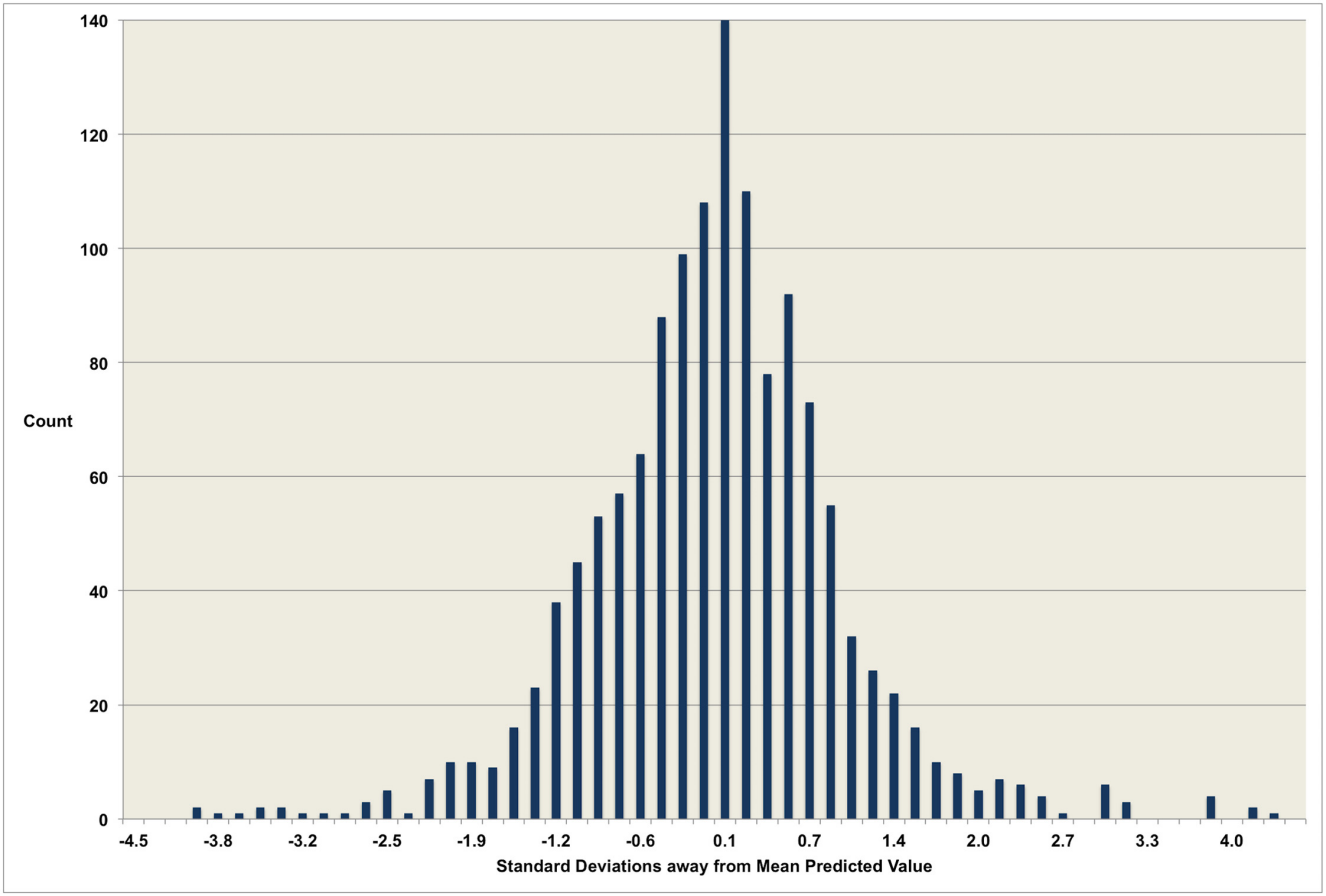
Residual analysis. The residuals are the variations in log-adjusted observed values from the log-adjusted age-related mean value predicted by the model. The residuals have excellent goodness of fit to an ideal Gaussian curve (r^2^ = 0.99). 71% of the residuals are within one standard deviations (SD) of the mean, 94% within 2 SD, and 98% within 3SD. The percentages for an ideal Gaussian distribution are 68%, 95% and 99% respectively.

1. the residuals of the test data were approximately normally distributed (i.e. the r^2^ for a normal Gaussian curve fitted to the residuals is close to one) (Fig 3); and
2. the mean square error for the cross-validation stages were (i) comparable to the overall mean square error and (ii) showed no trend towards overfit or underfit (Table 4).

**Table 3:** Model parameter values. Also given are the standard error and T statistic for the values, and the upper and lower 95% confidence limits for the values.

Parameter	Value	Std Error	T Value	95% CL lo	95% CL hi
a	0.217809	0.0134412	16.2045824	0.1914426	0.2441755
b	0.4488971	0.0008296	541.1232799	0.4472698	0.4505244
c	0.2611778	0.0131226	19.9029608	0.2354364	0.2869192
d	-0.0992615	0.0052107	-19.0495	-0.1094829	-0.0890401
e	-0.0390802	0.0058553	-6.6743402	-0.050566	-0.0275944
f	0.0058979	0.0009313	6.3332451	0.0040711	0.0077247
g	0.000399	0.0010997	0.3627855	-0.0017583	0.0025562
h	-0.0001055	0.0000489	-2.1546313	-0.0002015	-0.0000094
i	0.000102	0.0000569	1.7931277	-0.0000096	0.0002136
j	0.0000015	0.0000004	4.1251293	0.0000008	0.0000022

**Table 4:** Cross validation. MSE denotes mean square error. Training error and test error for the 5-folds of the data set. The first and third cross validation sets show slight overfit (error is higher for data not used to fit the model); the second and fourth sets show slight underfit. The overall error of 5.5% is similar to the error found at the cross validation stages.

K	Train MSE (%)	Test MSE (%)
1	5.2	6.6
2	5.7	5.3
3	5.7	5.9
4	5.7	5.1
**All data**	**5.5**	na

Approval was not required from an ethics committee or institutional review board since our research was limited to use of previously collected, non-identifiable data that has been published in peer reviewed journal, which is specifically excluded from Research Ethics Committee review by the National Research Ethics Service guidelines of the UK Health Research Agency [29]. Patient data from MRI scans were anonymized and de-identified by the researchers involved in their analysis. Written informed consent was obtained from participants (or next of kin/caregiver in the case of children) for their clinical data to be published in the studies that provided the data that we extracted to derive our normative model. No patient identifiable information was available to us at any stage of our investigation.

## Results

The validated model is a rational polynomial of the form

$$log_{10}\left(UV+1\right)=\;\frac{a+cx+ex^{2}+gx^{3}+ix^{4}}{1+bx+dx^{2}+fx^{3}+hx^{4}+jx^{5}}$$

where UV denotes uterine volume measured in cubic centimetres and x denotes age in years. Model coefficients a—j are given in Table 3, and relationship to the data given in Fig 3, with the model censored at age 40 due to sparse data for older ages (2 of 1,418 data values). The model has coefficient of determination r^2^ = 0.84 indicating that around 84% of the variation in uterine volumes in the healthy population up to age 40 is due to age alone. The r^2^ for the best-fitting LOESS model was 0.79, establishing the optimality of the single regression model in terms of goodness-of-fit. The residual plot for the validated model (Fig 3) shows a distribution close to the ideal Gaussian curve (r^2^ = 0.97). Moreover, the proportions of residuals within one, two and three standard deviations (respectively 71%, 94% and 98%) are close to the expected values for data with a perfect Gaussian distribution (respectively 68%, 95% and 99%).

**Fig 4:**
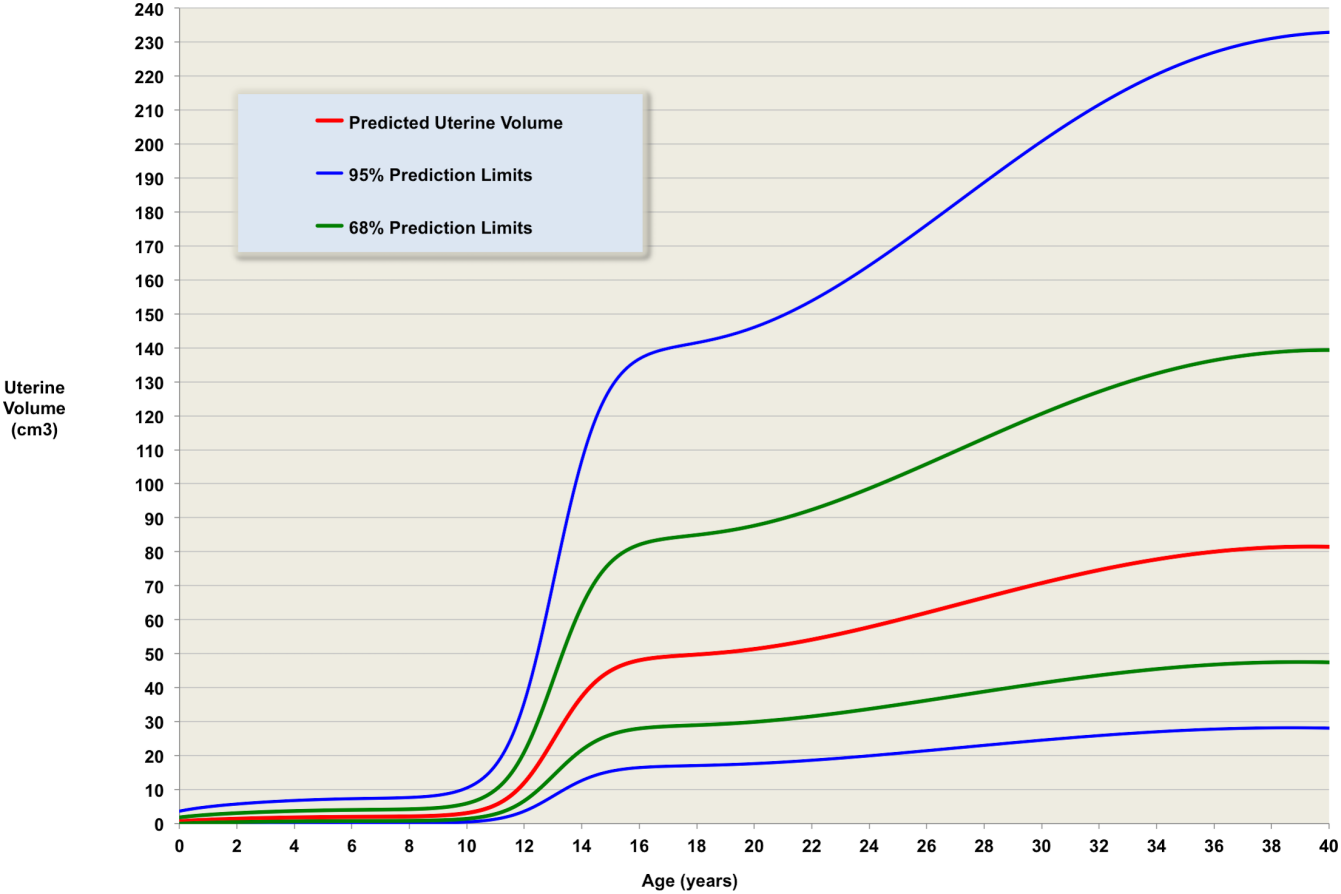
The validated model. Predicted uterine volume for ages from birth to 40 years, with one and two standard deviations prediction limits– 68% of measurements at a given age are expected to be between the green lines; 95% are expected to be between the blue lines.

**Fig 5:**
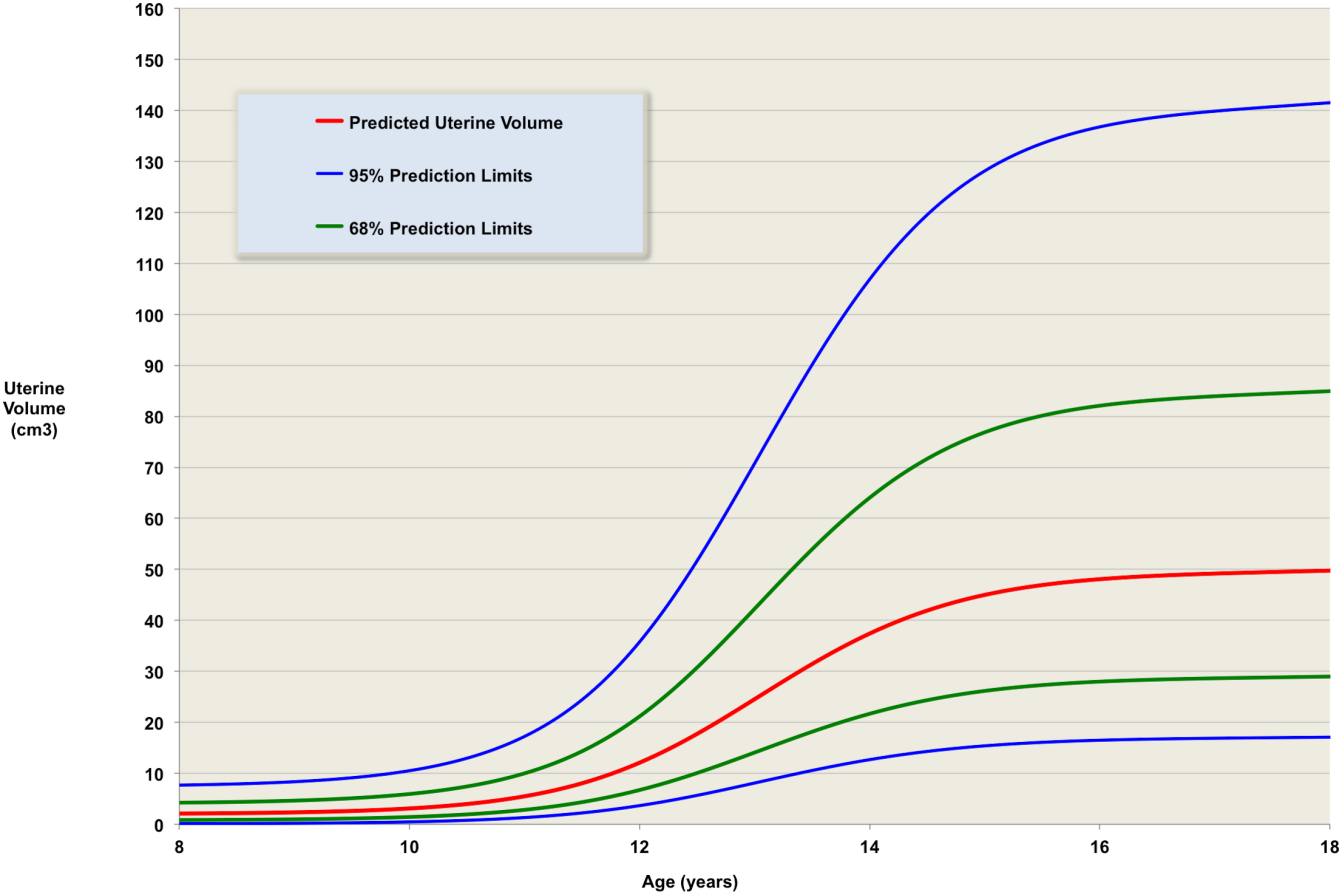
The validated model. Predicted uterine volume for ages from 8 to 18 years, with one and two standard deviations prediction limits– 68% of measurements at a given age are expected to be between the green lines; 95% are expected to be between the blue lines.

**Fig 6:**
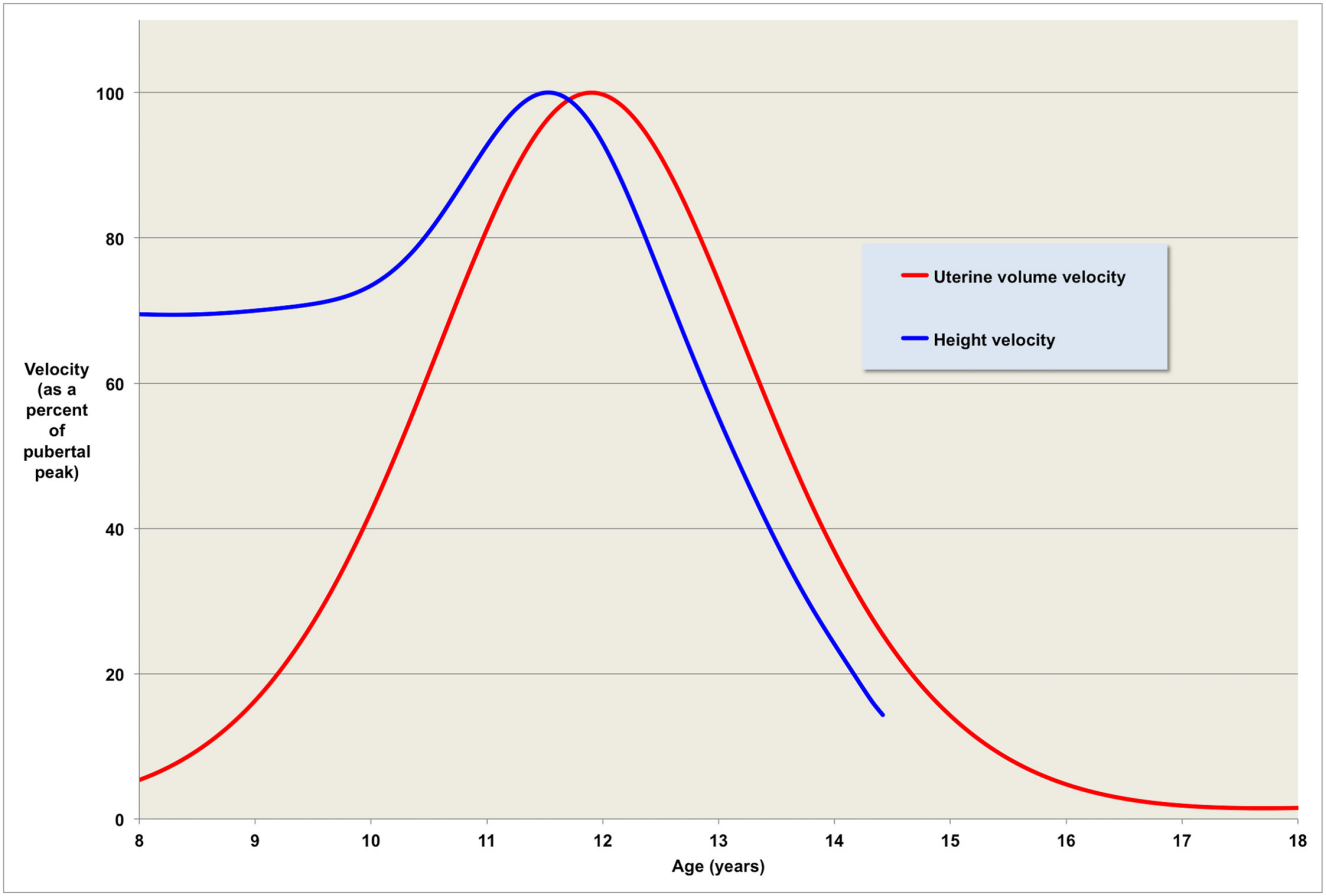
Velocity comparison. The first derivative of our uterine volume model as a percentage of the peak pubertal velocity is shown in red. For comparison, the height velocity for girls (based on data from [30]) is shown in blue.

Our log-unadjusted normative model (Fig 4) provides predicted average uterine volume for the entire age range up to age 40 years, together with normative ranges in terms of standard deviations away from age-related mean levels (Table 5). Fig 5 contains the same information for the restricted age range 8 to 18 years, emphasising the growth in uterine volumes at pubertal ages. A comparison of the velocities for height (taken from a standard reference [30]) and for our model of uterine volume is shown in Fig 6.

**Table 5:** Normative Uterine Volumes. Mean volumes (50th centile) in cm^3^ are given for ages 0 to 40. Also given are volumes at one standard deviation from the mean (16th and 84th centiles) and at two standard deviations from the mean (2.5 and 97.5 centiles).

Age	2.5 Centile	16th Centile	50th Centile	84th Centile	97.5 Centile
0	0	0.2	1.1	2.6	5
1	0	0.3	1.3	2.9	5.5
2	0	0.4	1.4	3.1	5.8
3	0	0.4	1.5	3.2	6.1
4	0	0.5	1.6	3.4	6.4
5	0	0.5	1.6	3.5	6.6
6	0	0.6	1.7	3.6	6.7
7	0	0.6	1.7	3.7	6.9
8	0	0.7	1.8	3.9	7.2
9	0.1	0.8	2.1	4.4	8
10	0.4	1.3	3	5.9	10.5
11	1.3	2.8	5.5	10.2	17.8
12	3.6	6.7	12.2	21.6	37
13	7.8	13.8	24.4	42.6	72.3
14	12.3	21.3	37.2	64.5	109
15	15	25.8	44.9	77.8	131.2
16	16.1	27.7	48.2	83.3	140.5
17	16.5	28.3	49.3	85.2	143.7
18	16.7	28.6	49.8	86.1	145.3
19	16.9	29	50.4	87.2	147
20	17.2	29.5	51.3	88.7	149.6
21	17.6	30.2	52.5	90.8	153.1
22	18.1	31.1	54	93.4	157.4
23	18.7	32.1	55.8	96.4	162.4
24	19.4	33.2	57.7	99.7	168.1
25	20.1	34.5	59.8	103.3	174.1
26	20.9	35.7	62	107.1	180.4
27	21.7	37	64.3	111	186.9
28	22.4	38.4	66.5	114.8	193.5
29	23.2	39.6	68.7	118.6	199.9
30	23.9	40.9	70.9	122.3	206
31	24.6	42	72.9	125.8	211.9
32	25.3	43.1	74.7	128.9	217.2
33	25.9	44.1	76.4	131.8	222.1
34	26.4	45	77.9	134.3	226.3
35	26.8	45.7	79.1	136.5	229.9
36	27.1	46.2	80.1	138.2	232.8
37	27.3	46.6	80.8	139.4	235.1
38	27.4	46.8	81.2	140.3	236.7
39	27.4	46.9	81.4	140.8	237.7
40	27.3	46.7	81.3	140.8	238.2

## Discussion

Using data-driven modelling and analysis of our own and others data, we have derived a normative model of uterine volume up to age 40 years. We have shown, in the healthy female, uterine volume does not increase in size during childhood, but thereafter there is a dramatic increase in size from age 10, presumably under the influence of puberty. The predicted volume of the uterus at age three years is 1.5 cm^3^ (68% prediction limit 1.5–3.2 cm^3^), whereas the predicted volume of the (post pubertal) uterus at age of 15 years is 25.8 cm^3^ (68% prediction limit 25.8–77.8 cm^3^).

Human growth during childhood has been described in terms of three biologically distinct components: infancy, childhood and puberty [31]. The infancy component is largely nutrition dependent, the childhood component is mostly dependent on growth hormone (GH) and the pubertal component depends on the synergism between sex steroids and GH. Uterine growth begins at the age of approximately 10 years closely in line with the onset of breast development and early pubertal development. In a large American study of 17,077 healthy girls, of whom 9.6% were African-American and 90.4% white, the mean age of the onset of breast development for white girls was 9.96 years (SD, 1.82) with menarche occurring on average at 12.88 years (SD, 1.20) [32].

We have also shown (Fig 6) that in normal females the age when maximum height velocity occurs during puberty is closely related to the maximum velocity of growth in volume of the uterus. The maximum height velocity precedes the maximum velocity of uterine growth by less than a year. Normal pubertal development in the female is characterised by a growth spurt that is concurrent with early breast development, and is blunted in the presence of GH insufficiency [33]. Although the data we have analysed or extracted is matched to age alone and not stage of pubertal development our study provides good evidence that the increase in uterine volume is concurrent with the onset of puberty and likely to be mediated by the production of sex steroids from the ovaries and GH from the pituitary.

Following puberty the steady rise in uterine volume is likely to be related to parity but we are not able to confirm this from the studies from which the data is extracted. It is a widely held belief that uterine size increases with subsequent pregnancies and some support for this hypothesis is provided by a study of umbilical cord length as a surrogate measure of uterine size [34]. What is apparent is that there is an increased variation in uterine volume with increasing age and while parity is likely to be a factor there may be other factors including the presence of small leiomyomata.

The assessment of uterine volume is important in the diagnosis and management of a number of conditions including Disorders of Sexual Development, vaginal bleeding in the prepubertal child, precocious puberty and delayed menstruation with or without secondary sexual characteristics. By providing a normative model of uterine volume for age pelvic ultrasound examination of the internal genitalia and the assessment of uterine volume will complement the GnRH test in the assessment of early and precocious puberty.

Nella *et al*. (2014) [35] have recently shown that isolated pre-pubertal vaginal bleeding is typically benign and self-limited when associated with normal prepubertal uterine findings on transabdominal USS. We have shown that TBI, probably through a direct effect on uterine blood supply, has a permanent and irreversible effect on uterine function. In these young patients with primary ovarian failure, uterine volume remained small despite three months treatment with physiological sex steroid therapy [7]. Others have shown that women with Turner syndrome treated with estrogen (of adequate dose and duration) may attain a normal, mature uterine volume, even at a late start of hormone replacement therapy and independently of karyotype [36].

We acknowledge that there is a relative paucity of data on uterine volume in girls under seven years of age in the published literature. As a direct result we cannot reliably rule out a small effect of the well characterised, but little understood, neonatal mini-puberty on uterine volume.

All of the acquired uterine volume data is from transabdominal ultrasound examinations in normal females, forming a representative sample of uterine volumes for ages from birth to 40 years. It is possible that bias has been introduced as a result of on ore more of the included studies reporting significantly lower (or higher) volumes for their age range(s). However, such a bias—if extreme enough—would be likely to produce an unrealistic model when combined with more accurate data. Since this has not been evident in our analyses, we are confident that any such bias is small. Our own data is from pelvic MRI examinations in normal females without a known endocrine, oncological or congenital disorder. Although we do not have a direct comparison in the same females between ultrasound obtained transabdominally and MRI assessment of uterine volume we have made the assumption that the derived uterine volumes are comparable. Evidence to support this assumption comes from a recent study, which reported an 89% correlation between transabdominal ultrasound and MRI assessments of uterine volume [37] with no evidence for systematic over- or underestimation of uterine volumes for either method for the entire range of volumes studied.

In summary we have shown that in the healthy female, uterine volume increases significantly in size under the influence of puberty, thereafter the steady rise in uterine volume is likely to be related to parity. The derivation of a validated normative model with age-related reference values for uterine volume from birth to age 40 years has important clinical applications in the assessment of females with Disorders of Sexual Development, vaginal bleeding in the prepubertal child, precocious puberty and delayed menstruation with or without secondary sexual characteristics.

## Supporting Information

S1 Dataset Combined uterine volume data and validation subsets. The Data worksheet contains our data and the volumes extracted from the published literature (Table 1). The Combined worksheet has the 1,418 age-uterine volume pairs (Table 1), with fixed zero values at conception. Raw values in cubic centimetres are log adjusted after adding 1 (so that zero volume is the same for adjusted and unadjusted data).
(XLS)
